# Reduction of the rocuronium-induced withdrawal reflex by MR13A10A, a generic rocuronium with a novel solution: A randomized, controlled study

**DOI:** 10.1371/journal.pone.0223947

**Published:** 2019-10-30

**Authors:** Masaru Shimizu, Fumimasa Amaya, Mao Kinoshita, Masaki Yamasaki, Isao Yokota, Teiji Sawa

**Affiliations:** 1 Department of Anesthesiology, Kyoto Prefectural University of Medicine, Kyoto, Japan; 2 Department of Pain Management and Palliative Care Medicine, Kyoto Prefectural University of Medicine, Kyoto, Japan; 3 Department of Biostatistics, Graduate School of Medicine, Hokkaido University, Sapporo, Japan; Imam Abdulrahman Bin Faisal University College of Medicine, SAUDI ARABIA

## Abstract

**Background:**

Rocuronium induces venous pain and the withdrawal reflex during injection. MR13A10A, generic rocuronium with a novel solution, reduced the injection-induced withdrawal reflex in rodents. We hypothesized that MR13A10A would reduce the frequency and severity of injection-induced withdrawal reflexes compared with original rocuronium during clinical anesthesia induction.

**Methods:**

This prospective, open (but assessor-blinded), randomized, controlled study was conducted at a single academic hospital. The assessor was blinded to the study condition in order to minimize observer bias. Participants were allocated to either MR13A10A or traditional formula groups by a blocked stratified randomization method. Participants in the MR13A10A group received MR13A10A, whereas the original rocuronium group received the same amount of original rocuronium. The primary outcome was presence of the withdrawal reflex after rocuronium injection. Severity of the withdrawal reflex, changes in blood pressure and heart rate, and the train of four (TOF) ratio were measured as secondary outcomes. The withdrawal reflex was assessed using a video recording in a blinded manner.

**Results:**

Of the 149 participants, 76 were allocated to the MR13A10A group and 73 to the original rocuronium group. The frequency of the withdrawal reflex was significantly lower with MR13A10A compared with original rocuronium (19.7% and 54.8% for MR13A10A and original rocuronium groups, respectively, p<0.001). The odds ratio adjusted for cannulation site, cannula size, induction agent and age was 6.27 (95% CI, 2.87, 13.73 p<0.001). Original rocuronium was an independent factor related to a higher post-treatment heart rate. The proportions of TOF ratios = 0 were similar between the two groups.

**Conclusion:**

The frequency and severity of the withdrawal reflex after injection were significantly reduced by using MR13A10A. MR13A10A might contribute to safe and less invasive anesthesia management.

## Introduction

Due to its favorable pharmacological characteristics, rocuronium is frequently used during the induction and maintenance of general anesthesia [1]. Patients who receive rocuronium while awake often complain of severe burning pain at the site of injection [2–4], associated with flexion of the wrist or arm [5]. Even when rocuronium is used after loss of consciousness, some patients reportedly exhibit spontaneous movement of the upper limb and recall injection pain after the surgery [6]. Rocuronium-induced spontaneous movement, i.e. withdrawal reflex, has been reported to occur in 50–84% of anesthetic inductions involving rocuronium [2, 7–9]. Several methods have been attempted to reduce the frequency of the withdrawal reflex after rocuronium injection. Non-pharmacological interventions include topical warming [7], cooling the reagent [10] and slowing the injection rate [11]. Pharmacological interventions include administration of opioids [8, 12, 13], lidocaine [8, 9, 13], ondansetron [8, 9], tramadol [8], magnesium sulfate [14], and ketamine [15, 16]. While most of these interventions successfully reduce the incidence of the withdrawal reflex, they require additional drug treatment and/or complicated procedures. MR13A10A, rocuronium dissolved with a novel solution, has been developed. Animal studies showed that MR13A10A had an identical pharmacological profile to rocuronium with the traditional formulation [17], but with a reduction in vascular irritability [18]. The Pharmaceutical and Medical Devices Agency (PMDA) in Japan approved marketing of MR13A10A as generic rocuronium in July 2017.

An animal study suggested that injection pain and the subsequent withdrawal reflex can be reduced by using MR13A10A in clinical settings. In this study, we hypothesized that MR13A10A could reduce the frequency of stimulation of the withdrawal reflex when compared to the original rocuronium. To investigate this hypothesis, a randomized, controlled study was performed to compare the frequency and severity of the withdrawal reflex induced by injection of MR13A10A as compared to the original rocuronium during induction of general anesthesia.

## Materials and methods

This prospective, open-labeled, randomized, controlled study was conducted at a single academic hospital. The assessor was blinded to minimize observer bias. This study was approved by the Institutional Review Board of Kyoto Prefectural University of Medicine (Certification No. ERB-C-653-2) and written informed consent was obtained from all subjects participating in the trial. Written, informed assent from the patients, as well as informed consent from their guardians, was obtained for participants younger than 20 years old. The trial was registered prior to patient enrollment at UMIN-CTR (UMIN000022300, http: https://upload.umin.ac.jp/cgi-open-bin/ctr_e/ctr_view.cgi?recptno=R000025693, Principal investigator: Fumimasa Amaya, MD, PhD, Date of registration: May 13 2016).

### Participants

Inclusion criteria were American Society for Anesthesiologists-physical status class I or II patients, aged between 6 months and 65 years, who required elective surgery under general anesthesia. Exclusion criteria were patients with neuromuscular diseases, history of allergy to thiopental and rocuronium, muscle weakness of the upper limb, obesity (body mass index >30 kg/m^2^) and refusal to participate in this study. Participants were stratified into two groups according to age ≥20 years and assigned to the MR13A10A or original rocuronium groups by the blocked, stratified randomization method (block size 4 or 6). A research assistant in the Center for Quality Assurance in Research and Development (CQARD) of Kyoto Prefectural University of Medicine performed participant assignment with computer-generated random numbers.

### Outcomes

The primary outcome was the frequency of stimulation of the withdrawal reflex after rocuronium injection. Severity of the withdrawal reflex, changes in blood pressure and heart rate, and the train of four (TOF) ratio were measured as secondary outcomes.

### Sample size

Sample size was calculated based on an estimated withdrawal reflex rate of 70% in the original rocuronium group and 45% in the MR13A10A group, based on our preliminary observations and previous studies [9, 13]. Sample size calculations indicated the need for 69 participants in one arm (overall n = 139) for analysis by Fisher’s exact test with α = 5% and 1-β = 80%. Considering drop-outs, the sample size for this study was determined to be 150 subjects.

### Protocol

None of the participants received pre-medication. After admission to the operating room, patients received standard hemodynamic monitoring for general anesthesia, including bispectral index monitoring and TOF monitoring (TOF-watch SX, Organon, Ireland). Inhalational induction was started with 5% sevoflurane for participants younger than 14 years old, and standard induction with propofol (1–2 mg/kg body weight) or thiopental (3–5 mg/kg body weight) was performed in participants 14 years old or older. Intravenous cannulation was achieved on the dorsal part of the hand or dorsal side of the forearm. Rocuronium (0.9 mg/kg) was injected after confirming loss of consciousness by loss of the eyelash reflex and a bispectral index less than 60. The MR13A10A group received MR13A10A, and the original rocuronium group received original rocuronium. Intravenous opioid injection and tracheal intubation were performed after the observation period.

### Rocuronium formula

MR13A10A (Rocuronium Bromide intravenous solution®, Maruishi Pharmaceutical Co. Ltd., Osaka, Japan) contained 1% rocuronium bromide, 0.5% sodium chloride, and 0.55% glycine. The solution was adjusted to pH 3. The osmotic pressure ratio was 1 [19]. The original rocuronium (ZEMURON®, Organon, NJ, USA) contained 1% rocuronium bromide with sodium acetate and sodium chloride as the buffer solution, adjusted with acetic acid to a pH of 4. MR13A10A was provided by Maruishi Pharmaceutical Co. Ltd. The traditional formula was purchased from MSD Japan (Tokyo, Japan). The Pharmaceuticals and Medical Devices Agency (PMDA) approved MR13A10A for marketing in June 2017, and the present study began afterwards. Informed consent was obtained from the participants by explaining this.

### Observations

The upper arm and body of the participants were observed for three minutes after the rocuronium injection using a video camera system (HDR-AS300, Sony Co., Tokyo, Japan). The camera was set at 1 m above the patient’s body to obtain whole-body images. It captured movies of size 1920 × 1080 with a frame rate of 30 fps in MP4 format. An assessor, one of the authors (MY), who was blinded to the experimental condition, checked the video recordings on a Macintosh computer (MacBook Air, Apple Inc., Cupertino, CA) and assessed the withdrawal reflex based on the scores established in a previous study with modification (Table 1) [9]. A score of 0, “unable to determine arm movement”, was added. In order to perform standardized assessments, one experimenter (YM) assessed all the participants. Scores 0 and 1 were defined as “response undetected”, whereas scores 2, 3, and 4 were defined as “response detected”. Hemodynamic parameters, including mean blood pressure and heart rate, and the TOF ratio were recorded prior to and three minutes after the rocuronium injection. Caregivers of the participants were blinded to the study.

**Table 1:** Withdrawal reflex score.

0	Unable to determine	
1	No response	
2	Wrist	movement at the wrist only
3	Elbow/Shoulder	withdrawal involving arm only
4	Generalized	withdrawal or movement in more than one extremity, cough, or breath-holding

### Statistical analysis

Analysis was performed using the intention to treat principle and with the full analysis set. Comparisons of two continuous outcomes were performed by Student’s *t*-test or Mann-Whitney’s U test, as appropriate. Comparison of the proportion of patients who exhibited the withdrawal reflex was performed by Fisher’s exact test and estimated odds ratios. Since vascular diameter and participant age are reportedly associated with the withdrawal reflex after rocuronium injection [20, 21], logistic regression analysis was also performed to obtain adjusted odds ratios with adjustment for these factors. Comparison of the severity of the withdrawal reflex was performed by the Mantel-Haenszel test for trend. Blood pressure and heart rate after the rocuronium injection were analyzed by linear regression analysis adjusted for the baseline values (blood pressure and heart rate before injection), participant age, and rocuronium formula. Comparison of the proportion of patients with TOF ratios of zero after rocuronium injection was performed by Fisher’s exact test. All statistical analyses were performed using STATA version 14 (StataCorp LLC, College Station, TX). A p value <0.05 was considered significant. All of the data in this study were under the management of CQARD. The sponsoring party was not involved in data collection, management, or analysis.

## Results

**Fig 1:**
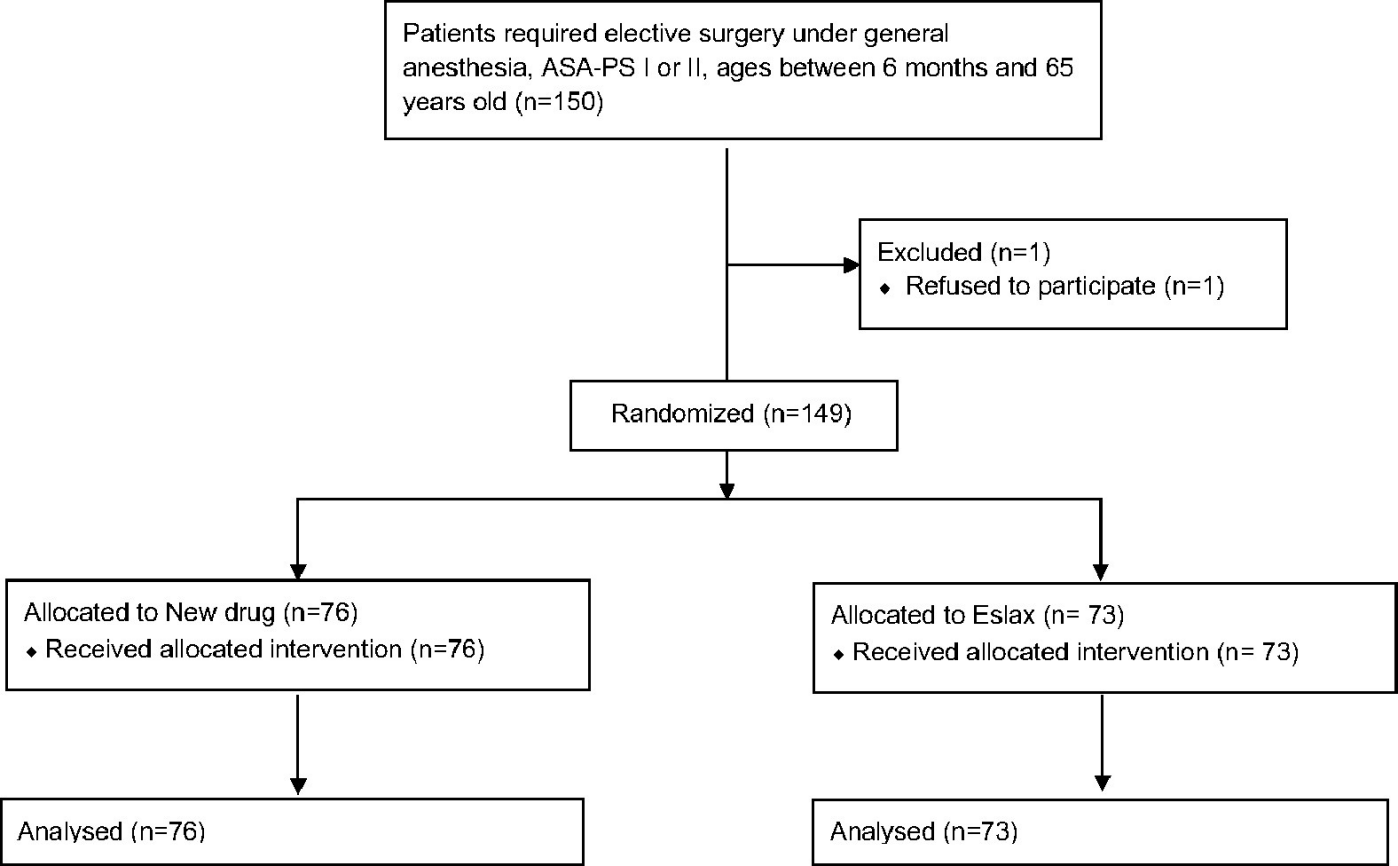
Flow diagram of this study.

A total of 150 patients were enrolled in this study from September 2017 to June 2018, one of whom refused participation after allocation. Therefore, 149 participants (76 and 73 in the novel and traditional formula groups, respectively) were analyzed. Fig 1 presents a flow diagram of the study. Table 2 shows the demographic data of the participants. There were no significant differences in demographic data between the two groups. All anesthesia inductions of participants 14 years old or older were performed with 5 mg/kg of thiopental.

**Table 2:** Characteristics of the patients.

Characteristics	MR13A10A (n = 76)	Original rocuronium (n = 73)
Age, years ^a^	46.0 (30.8, 56.0)	47.0 (35.0, 56.0)
Among those aged ≥20 y ^a^	47.0 (38.0, 57.0)	48.5 (40.4, 57.8)
≥20 y, No. (%)	69 (90.8)	66 (90.4)
Male, No. (%)	37 (48.7)	26 (35.6)
Height, (cm) ^a^	163.3 (156.5, 170.2)	159.5 (155.5, 167.0)
Weight, (kg) ^a^	58.0 (50.6, 67.0)	57.6 (48.5, 65.0)
BMI, (kg/m^2^) ^a^	21.7 (20.0, 23.9)	21.7 (18.7, 23.7)
ASA, No. (%)		
I	31 (40.8)	36 (49.3)
II	45 (59.2)	37 (50.7)
IV cannula size No. (%)		
Forearm	16 (21.1)	24 (32.9)
Dorsum	60 (78.9)	49 (67.1)

^a^ median (interquartile range 25–75%), Mann-Whitney’s U test, BMI, Body Mass Index; ASAPS, American Association of Anesthesiologists physical status.

Movement of the upper arm could not be determined in 4 participants from the video recording images (2 participants in both groups) and was scored as 0. They were included in the “response undetected group”. Table 3 demonstrates the proportion of patients with or without withdrawal reflex stimulation after rocuronium injection. The percentage of participants who exhibited the withdrawal response was 19.7% and 57.8% for MR13A10A and original rocuronium, respectively (p<0.001). The percentage of participants with the withdrawal response was 17.4% and 54.5% (p<0.001), respectively, when the analysis was limited to participants older than 20 years (Table 3, lower part). Unadjusted and adjusted odds ratios for arm movement after drug injection are shown in Table 4. A similar difference in the proportion of patients with a withdrawal reflex was observed when the 4 participants with a score of 0 were excluded from the analysis (S1 Table). Response scores of the withdrawal reflex in both groups are shown in S2 Table. The distribution of the severity of withdrawal reflexes was significantly different between the two groups.

**Table 3:** Withdrawal response associated with injection of MR13A10A and original rocuronium.

All participants	MR13A10A	Original rocuronium
Response undetected, No. (%)	61 (80.3)	33 (45.2)
Response detected, No. (%)	15 (19.7)**	40 (54.8)
**Participants older than 20 years**	MR13A10A	Original rocuronium
Response undetected, No. (%)	57 (82.6)	30 (45.5)
Response detected, No. (%)	12 (17.4)**	36 (54.5)

**p<0.001 by Fisher’s exact test.

**Table 4:** Unadjusted and adjusted odds ratio for arm movements after injection of MR13A10A or original rocuronium.

	Odds ratio (95% CI)	p-value
Unadjusted odds ratio	4.93 (2.38, 10.22)	< 0.001
Adjusted odds ratio*	6.40 (2.91, 14.08)	< 0.001
Adjusted odds ratio**	6.27 (2.87, 13.73)	<0.001

*Adjusted by location of cannulation site, cannula size, induction agent, and age.

**Adjusted by location of cannula site, cannula size, and induction agent.

Mean blood pressure and heart rate after the drug injection are shown in Table 5. Multiple linear regression analysis demonstrated that rocuronium solution was an independent factor related to post-treatment heart rate when adjusted by age and pre-treatment heart rate (Table 6). The percentages of participants who showed TOF ratios of zero were similar between the two groups (52.6% and 50.7% for MR13A10A and original rocuronium, respectively, Table 7). No severe adverse effects were recorded, except for skin redness in three participants (1 and 2 for MR13A10A and original rocuronium, respectively).

**Table 5:** Hemodynamics after injection of MR13A10A and original rocuronium.

	MR13A10A	Original rocuronium	p-value*
Mean blood pressure	88.5 ± 14.7	93.8± 21.7	0.08
Heart rate	84.0 ± 11.6	89.9 ± 14.3	<0.01

Mean ± SD,

*Student’s *t*-test.

**Table 6:** Multiple linear regression analysis of post-treatment hemodynamics by the rocuronium formulation.

	Coefficient (95% CI)	p-value
Mean blood pressure	5.08 (-0.14, 10.17)	0.051
Heart rate	4.75 (0.85, 8.64)	< 0.017

Multiple linear regression analysis adjusted by age and baseline value (mean blood pressure and heart rate prior to the drug injection).

**Table 7:** TOF scores 3 minutes after injection of MR13A10A and original rocuronium.

	MR13A10A	Original rocuronium
0	40 (52.6)	37 (50.7)
1, 2, 3, 4	36 (47.4)	36 (49.3)

p = 0.87 by Fisher’s exact test, No (%).

## Discussion

In the present study, there was a lower incidence of withdrawal responses with the use of MR13A10A, a generic rocuronium with a novel solution, demonstrating that the novel solution reduced rocuronium-induced vascular pain. The efficacy of MR13A10A in reducing the frequency of withdrawal responses was as high as that of the most effective treatment strategy of lidocaine injection under tourniquet application previously shown [8]. MR13A10A is less expensive than original rocuronium in Japan ($5 and $7.5, respectively). The cost of original rocuronium is 23$ in the US. The cost of MR13A10 in the US or Europe is unknown as this drug is not used in these regions now, but might be similar to the generic rocuronium (varies from 7.5$ to 20$ in the US). Therefore, use of MR13A10A enables reduction of the withdrawal response rate without additional cost. Another generic rocuronium approved in the United States may not have this effect, since their solution is the same as the original rocuronium.

Many papers have reported considerable numbers of patients who exhibit the withdrawal reflex after rocuronium injection during the induction of general anesthesia. Since rocuronium-induced pain is due to activation of C-fiber neurons [22], vascular pain is likely to induce the withdrawal reflex. Dilution of rocuronium reduces the painful sensation, suggesting that the concentration of rocuronium or buffer salt determines vascular irritability [23]. Jimbo et al developed MR13A10A with substitution of the acetate buffer by glycine and hydrochloric acid, and observed that the novel solution of MR13A10A greatly reduced vascular pain-associated behavior in rats [18]. In the present study, the withdrawal reflex was significantly lower after injection of MR13A10A compared to the traditional formula. The efficacy of MR13A10A in preventing reflex withdrawal was comparable or much greater than that previously reported with preventive interventions [24].

Since vascular diameter is reportedly associated with the occurrence of the withdrawal reflex after rocuronium injection [20, 21], multivariate analysis was performed to exclude the effects of catheter size and location on vessel diameter at the access site. The adjusted odds ratio was 6.40 (95%CI: 2.91, 14.08), confirming a significantly lower incidence of the withdrawal reflex using MR13A10A.

However, the withdrawal reflex did not completely disappear in patients who received MR13A10A; 20% of participants in the novel formula group displayed the withdrawal reflex. MR13A10A therefore may cause vascular irritability to some extent. In the present study, all adult participants were given thiopental as an induction drug, because thiopental causes less vascular pain than propofol. However, we cannot completely exclude the possibility that the induction agent might have induced the withdrawal reflex.

Nociceptive stimulation can activate the sympathetic nervous system and increase blood pressure or heart rate. Original rocuronium was found to be the independent factor associated with a higher heart rate. However, there were no increases in mean blood pressure 3 minutes after the injection of rocuronium. The vasodilatory effect of thiopental/sevoflurane might have affected the blood pressure. TOF-watch data demonstrated that the potencies of the muscle relaxant effects of rocuronium were similar for the two formulations.

The present study has several limitations to note. Participant age was not limited in order to study the effect of MR13A10A in the general population. Further, block randomization prevented deviation of the participant generation. However, one cannot completely exclude the effects of different withdrawal reflex rates and different methods of anesthesia induction between children and adults on the results of this study. The number of children participants in both groups was smaller than had been expected. Therefore, a secondary analysis to see the effect of MR13A10A on children was not performed. Further study including a larger number of children participants will be needed to provide direct evidence. The definition of adult as 20 years old or older was decided based on the legal age of adulthood in Japan at that time. There are several cut points that distinguish adults from children. Use of a different definition may have had some effect on the present result. Next, due to technical difficulties, the anesthesiologists in charge were not blinded to the experimental condition. However, one experimenter who was blinded to the experimental conditions assessed for withdrawal reflexes. Strict judgement about the existence of arm movement made it difficult to determine the presence or absence of the withdrawal reflex in 4 participants. Another limitation of the present study is that the frequency of withdrawal reflex stimulation could have been affected by the thiopental or sevoflurane used for anesthesia induction in both groups [25, 26].

## Conclusions

In conclusion, reductions of the frequency and severity of withdrawal reflexes after rocuronium injection were observed by using MR13A10A. This, together with previous animal studies showing the lower tissue irritability of this formula, suggest that rocuronium-induced vascular pain can be minimized by using this formula. Use of MR13A10A is potentially beneficial in terms of safe and less invasive management of general anesthesia.

## Supporting information

S1 Table (DOCX)

S2 Table (DOCX)

S1 Checklist CONSORT 2010 checklist. (DOC)

S1 Protocol study_protocol_English. (PDF)

S2 Protocol study_protocol_Japanese. (PDF)

S3 Protocol study protocol_English_full. (DOCX)
