# Supplementary material for: Reduction of the rocuronium-induced withdrawal reflex by MR13A10A, a generic rocuronium with a novel solution: A randomized, controlled study
Source: PLoS One. 2019 Oct 30;14(10):e0223947. doi: 10.1371/journal.pone.0223947 (PMC6821093; doi:10.1371/journal.pone.0223947)
Supplement: S1 Table — (DOCX) [file pone.0223947.s001.docx]

**S1 Table Withdrawal response associated with injection of the novel and traditional rocuronium formulations**

**All participants**

|  | MR13A10A | Original rocuronium |
| --- | --- | --- |
| No response, No. (%) | 59 (79.7) | 31 (43.7) |
| Any Response, No. (%) | 15 (20.3) | 40 (56.3) |

p<0.001 by Fisher’s exact test

**Participants older than 20 years**

|  | MR13A10A | Original rocuronium |
| --- | --- | --- |
| No response, No. (%) | 55 (82.1) | 28 (43.7) |
| Any Response, No. (%) | 12 (17.9) | 36 (56.3) |

p<0.001 by Fisher’s exact test
