# Supplementary material for: Reduction of the rocuronium-induced withdrawal reflex by MR13A10A, a generic rocuronium with a novel solution: A randomized, controlled study
Source: PLoS One. 2019 Oct 30;14(10):e0223947. doi: 10.1371/journal.pone.0223947 (PMC6821093; doi:10.1371/journal.pone.0223947)
Supplement: S2 Table — (DOCX) [file pone.0223947.s002.docx]

**S2 Table Response score after injection of the novel or traditional rocuronium formulation**

|  | MR13A10A (n=74) | Original rocuronium (n=71) |
| --- | --- | --- |
| 1 | 59 (79.7) | 31 (43.7) |
| 2 | 1 (1.4) | 6 (8.5) |
| 3 | 11 (14.9) | 25 (35.2) |
| 4 | 3 (4.1) | 9 (12.7) |

p<0.001 by Mantel–Haenszel test for trend, No (%).
