# Supplementary material for: Reduction of the rocuronium-induced withdrawal reflex by MR13A10A, a generic rocuronium with a novel solution: A randomized, controlled study
Source: PLoS One. 2019 Oct 30;14(10):e0223947. doi: 10.1371/journal.pone.0223947 (PMC6821093; doi:10.1371/journal.pone.0223947)
Supplement: S1 Protocol — (PDF) [file pone.0223947.s004.pdf]

Study protocol

(1) Study design

This was an active-controlled trial, in which a parallel group comparison of a novel rocuronium formulation and the traditional rocuronium formulation was conducted. Both formulations were used within approved indications (legal use). The two formulations were allocated in a random, open-label fashion, but the research team members evaluating the primary outcome were blinded to group allocation.

(2) Sample size and its rationale

1. Sample size: Researchers' university: 150 cases; total: 150 cases
2. Rationale: From the results of previous and pilot studies, the withdrawal response incidence in comparison groups was assumed to be 70% and the expected withdrawal response incidence in the study drug group is 45%. Based on Fisher's exact test, with an assumed significance level of 2.5% (one-sided) and a power of 80%, the sample size was calculated to be 69 in each group, with a total of 138 people.

Considering drop-outs, we determined the sample size as 150 subjects in this study.

(3) Methods

1. Samples/information used

Of the patients who underwent scheduled surgery under general anesthesia, patients/guardians from whom written consent for participation in this clinical study was obtained and those who were between the ages of 6 months and 65 years on the day consent was obtained were accepted as subjects, with no distinction by sex.

Patients excluded due to safety concerns:

- Patients with myopathy
- Patients with a history of hypersensitivity to rocuronium
- Patients with a history of hypersensitivity to both propofol and thiopental
- Patients with decreased upper limb muscle strength
- Obese patients (BMI ≥30)

2. Endpoints

Primary endpoint:

The primary endpoint was presence or absence of a withdrawal response after administration of the drugs. Scores from 2 to 4 points on the scale of Ahmad et al., that is, when movement of the arms is seen, were defined as withdrawal response[1]. A score of 1 on Ahmad's scale, that is, absence of movement, was defined as no withdrawal response.

Secondary endpoints

- Magnitude of withdrawal response assessed using the scale of Ahmad et al.
- Blood pressure before and after drug administration
- Change in heart rate before and after drug administration

3. Observation and test items

Drug administration method

After entering the operation room, the subjects' vital signs were monitored, the same as

during regular general anesthesia, using an electrocardiograph, pulse oximeter, neuromuscular monitor, and sphygmomanometer.

Anesthesia was induced with intravenous administration of propofol (1-2 mg/kg body weight) or thiopental (3-5 mg/kg body weight). In cases in which standard induction was difficult, anesthesia was induced with inhalation of the anesthetic gas sevoflurane (mixture of 5% concentration in respiratory gas). Loss of consciousness was confirmed by disappearance of the patient's eyelash reflex, following which the study was commenced. The rocuronium formulation was administered at the dose of 0.9 mg/kg body weight. In the traditional formation group, traditional formula rocuronium was administered, and the novel formula rocuronium was administered in the novel formation group.

Observation of physical changes

The parameters observed were withdrawal response of the arm in which the infusion needle was inserted and changes in heart rate and blood pressure. The arm observations were started just before the administration of rocuronium and made for 3 minutes after its administration. The upper body was imaged with a video camera centered on the arm with the infusion needle, and a research member blinded to the drug content evaluated the withdrawal response as seen in the video camera images and scored it according to its magnitude. The evaluations were done by the same researcher throughout the study to avoid inter-rater bias. Criteria used in previous published reports on vascular pain due to rocuronium injection were employed in the scoring, with a score of 2 or more taken to indicate the presence of a withdrawal response. For the changes in heart rate and blood pressure, the values just before and for 3 minutes after administration were compared with reference to the vital sign monitor records. Observation of the study subjects ended at the point when the 3-minute observations were completed.

Data collection items

The following data items were collected in this study.

• Subject characteristics: Sex, date of birth, height (cm), weight (kg), date consent was obtained, legal representative, primary disease leading to surgery, history of drug hypersensitivity.

• Evaluation at anesthesia: Administered drugs (traditional formation rocuronium, novel formation rocuronium), dosage, administration start date, presence of the arm withdrawal response and magnitude of the response (as evaluated by the blinded research member), heart rate (just before and 3 minutes after administration), systolic and diastolic blood pressure (just before and 3 minutes after administration), TOF ratio according to the muscle relaxation monitor (just before and 3 minutes after administration).

• Adverse events: Type of event, date of occurrence, seriousness, whether there was a causative association, outcome, date outcome was confirmed.

• Drop out information: Day of discontinuation, reason for discontinuation.

4. Statistical analysis method

The population to be analyzed was taken to be the full analysis set using the assigned treatment group in accordance with the principle of intention-to-treat. Fisher's exact test

was performed for the primary outcome of whether or not there was a withdrawal response. Mantel's trend test was performed for the secondary outcome of the magnitude of the withdrawal response. Paired t-tests were performed for changes in blood pressure and heart rate before and after drug administration.
For the above analysis method, the details are described in the statistical analysis plan specified at the time of unblinding. However, other statistical method would be used in case that adjusted analysis by using participants' age or other factors would be preferable.

5. Drug/medical device overview
Information on the drug used in the trial is summarized below.
Name of drug used: Rocuronium bromide intravenous solution
Study drugs: Traditional formation rocuronium, 50 mg/5.0 mL (MSD K.K.), novel formation rocuronium (Maruishi Pharmaceutical Co., Ltd.)
Approval status in study subjects: Within legal use.
Indications: Muscle relaxation during anesthesia, muscle relaxation during tracheal intubation
Storage: Store at 2–8°C
Clinically significant adverse reactions: Shock, anaphylaxis, prolonged respiratory depression, bronchospasm
Other precautions: There are reports of hearing loss when other nondepolarizing muscle relaxants were administered in the same manner in critically ill neonates or infants.

1. Shevchenko Y, Jocson JC, McRae VA, Stayer SA, Schwartz RE, Rehman M, et al. The use of lidocaine for preventing the withdrawal associated with the injection of rocuronium in children and adolescents. Anesth Analg. 1999;88(4):746-8.

This protocol was approved by IRB of Kyoto Prefectural University of Medicine (May 19, 2017).
