# Supplementary material for: Reduction of the rocuronium-induced withdrawal reflex by MR13A10A, a generic rocuronium with a novel solution: A randomized, controlled study
Source: PLoS One. 2019 Oct 30;14(10):e0223947. doi: 10.1371/journal.pone.0223947 (PMC6821093; doi:10.1371/journal.pone.0223947)
Supplement: S2 Protocol — (PDF) [file pone.0223947.s005.pdf]

研究の方法

（1）研究のデザイン

治療介入研究

新規ロクロニウム製剤と従来使用されているエスラックス製剤の並行群間比較をおこなう実薬対照試験である。両薬剤とも承認適応症範囲内の試験となる。両薬剤は無作為・非盲検割り付けで行うが、プライマリーアウトカムの評価者は盲検化する。

（2）予定症例数とその設定根拠

①予定症例数：本学　150　例・全体　150　例

②設定根拠：先行研究およびパイロット研究の結果より、対照薬群での逃避行動割合を 70%、試験薬群での期待逃避行動割合を45%とし、Fisherの正確検定に基づき、有意水準を片側2.5%、検出力を 80%としたところ、片群 69 例、合計 138 例のサンプルサイズが算出された。

そこで本研究では、若干の解析除外例が生じることを考慮し、合計 150 例をサンプルサイズとして設定する。

（3）方法

①使用する試料・情報等

□ ア試料（内容：　　　　　　　　）

■ イ情報（内容：診療録　　　　　　）

□ ウその他（内容：　　　　　　　）

全身麻酔により定期手術を受ける患者のうち、本臨床研究への参加に関して文書による同意を取得し、同意取得日時点で 6 ヶ月以上 65 歳以下である患者を対象とする。性別を問わない。

除外患者

・筋疾患を有する患者

・エスラックスに対する過敏性の既往のある患者

・プロポフォールとチオペンタールの両者に過敏性の既往のある患者

・上腕の筋力が低下している患者

・肥満患者（BMI30 以上）

これらの患者は、安全性の観点から除外する。

②評価項目

主要評価項目：

主要評価項目は薬剤投与後の逃避行動の有無とする。Ahmad ら(2)の尺度における 2 点から 4 点、すなわち上肢の動きがみられた場合に逃避行動有りと定義し、同尺度における 1 点、すなわち動きなしの場合に逃避行動無しと定義する。

副次評価項目・

・Ahmad らの尺度により評価した逃避行動の程度
・投与前後の血圧
・投与前後の心拍数変化
③観察及び検査項目
（薬剤投与方法）
手術室入室後、バイタルサインのモニタリングを通常の全身麻酔と同様に、心電図、パルスオキシメーター、筋弛緩モニターおよび血圧計を用いて行う。
麻酔の導入は静脈麻酔薬プロポフォール（体重 1kg あたり 1-2mg）またはチオペンタール（体重 1kg あたり 3-5mg）の静脈内投与により行う。なお、点滴が困難な場合はガス麻酔薬セボフルラン（呼吸ガスに 5%濃度を混合）を吸入させて麻酔を導入する。眉毛反射の消失により患者の入眠を確認し、調査を開始する。ロクロニウム製剤を体重 1kg あたり 0.9mg 投与する。エスラックス群にはロクロニウム製剤としてエスラックスを投与し、新薬群では新薬を投与する。エスラックスは製造販売の承認をうけている。新薬の製造販売承認は 2016 年 8 月に許可される予定であり、許諾がない限り本研究は行わない。新薬はロクロニウム後発品であり、その効能効果はエスラックスと同一である。
（身体的変化の観察）
観察項目は、点滴が刺入された上肢の逃避行動ならびに心拍数と血圧の変化である。上肢の観察はエスラックス投与直前から開始し、投与後 3 分間まで行う。点滴刺入側の上肢を中心とした上半身をビデオカメラで撮影し、投薬内容について盲検化された研究分担者がビデオカメラの映像をもとに逃避行動を評価し、その程度に応じてスコアリングする。評価は研究を通じて同一の分担者が行うことで評価者間のバイアスが生じないようにする。スコアリングは、ロクロニウムによる血管痛について過去に出版公表された論文で使用された基準を利用し、2 点以上の場合「逃避行動あり」と判定する[1]。心拍数と血圧の変化はバイタルサインモニターの記録を参照し、投与直前と投与後 3 分間の値を比較する。3 分間の観察終了時点で対象患者の研究は終了する。
（データ収集項目）
本研究では以下のデータ項目を収集する。
・被験者背景：性別、生年月日、身長（cm）、体重（kg）、同意取得年月日、代諾者、手術に至った原疾患、薬物過敏症既往
・麻酔時評価：投与対象薬剤（エスラックス・新薬）、投与量、投与開始日、上肢逃避行動の有無、程度（盲検化された分担者による評価結果）、心拍数（投与直前・投与後 3 分後）、血圧 SBP、DBP（投与直前・投与後 3 分後）、筋弛緩モニターTOF 比（投与直前・投与 3 分後）
・有害事象：事象名、発現日、重篤度、因果関係有無、転帰、転帰確認日
・中止時：中止日、中止の理由

④統計解析の方法

解析対象集団は Intention-To-Treat の原則に従い、割付群を用いた Full Analysis Set とする。プライマリーアウトカムである逃避行動の有無に対し、Fisher の正確検定を行う。セカンダリーアウトカムである逃避行動の程度については Mantel 傾向検定を行う。投与前後の血圧および心拍数変化に対しては、対応のある t 検定を行う。

以上の解析手法について、盲検化解除までに定める統計解析計画書にてその詳細を記す。ただし、割り付けに用いた層別因子である年齢や必要と思われる因子を調整した解析を主たる解析として用いる場合、上記の解析手法と異なる解析を用いる場合がある。

⑤医薬品・医療機器の概要等

本試験に用いる薬剤情報の要約を以下に記載する。詳細については、最新の添付文書を参照のこと

使用する医薬品名：ロクロニウム臭化物注射液

試験薬：エスラックス静注 50mg/5.0mL（既存薬 MSD）、新規後発品（丸石製薬株式会社）

研究対象における承認状況：適応内

効能：麻酔時の筋弛緩、気管挿管時の筋弛緩

貯法：2～8℃に保存（毒薬）

重大な副作用：ショック、アナフィラキシー、遷延性呼吸抑制、気管支痙攣

その他の注意：他の非脱分極性筋弛緩剤で、同様の投与を重症の新生児又は乳児に行った際に、難聴を生じたとの報告がある。

1. Shevchenko Y, Jocson JC, McRae VA, Stayer SA, Schwartz RE, Rehman M, et al. The use of lidocaine for preventing the withdrawal associated with the injection of rocuronium in children and adolescents. Anesth Analg. 1999;88(4):746-8.

2017 年 5 月 19 日　京都府立医科大学医学倫理審査委員会　承認
