# Supplementary material for: Reduction of the rocuronium-induced withdrawal reflex by MR13A10A, a generic rocuronium with a novel solution: A randomized, controlled study
Source: PLoS One. 2019 Oct 30;14(10):e0223947. doi: 10.1371/journal.pone.0223947 (PMC6821093; doi:10.1371/journal.pone.0223947)
Supplement: S3 Protocol — (DOCX) [file pone.0223947.s006.docx]

1. **Title of study**

**Effect of a new rocuronium formulation in reducing injection pain**

1. Objectives and significance

Objectives

We will investigate whether the injection pain associated with induction of anesthesia is milder when using a new rocuronium formulation than when using Eslax. The primary outcome is whether or not there is a withdrawal response of the upper limb after injection of the drugs. The secondary outcomes are the magnitude of the withdrawal response and changes in blood pressure and heart rate before and after injection of the drugs.

Significance

Rocuronium is a non-depolarizing muscle relaxant that is widely used during the induction of general anesthesia due to its superior muscle relaxant effect and its high level of safety owing to the existence of a specific antagonist.

However, the rocuronium formulation currently sold in Japan (generic name: Eslax) causes transient vascular irritation at the time of intravenous injection. Since this irritation is transient and rocuronium is injected after the onset of sleep, it is rare for the patient to feel the irritation at the time of injection as pain or for vascular pain to occur after the conclusion of surgery. However, there are instances when the irritation can be problematic in terms of anesthesia management, such as when the patient cannot be kept still due to a reflexive withdrawal response or when heart rate and blood pressure increase as a consequence. Therefore, development and clinical application of a rocuronium formulation that is less irritating on injection could improve the quality and safety of general anesthesia.

1. Basis and background of scientific rationale

The new rocuronium formulation developed by Maruishi Pharmaceutical Co., Ltd. (new drug) has been shown in animal experiments to be less irritating to the blood vessel during intravenous injection than Eslax. This effect was achieved by switching the buffer contained in the injection solvent from sodium acetate to glycine. This study is being conducted because there have been no studies on whether the new drug reduces the injection pain of rocuronium when used in general anesthesia in clinical settings.

1. Methods

4.1. Study design

This was an active-controlled trial, in which a parallel group comparison of a novel rocuronium formulation and the traditional rocuronium formulation was conducted. Both formulations were used within approved indications (legal use). The two formulations were allocated in a random, open-label fashion, but the research team members evaluating the primary outcome were blinded to group allocation.

4.2. Sample size and its rationale

4.2.1. Sample size: Researchers’ university: 150 cases; total: 150 cases

4.2.2. Rationale: From the results of previous and pilot studies, the withdrawal response incidence in comparison groups was assumed to be 70% and the expected withdrawal response incidence in the study drug group is 45%. Based on Fisher’s exact test, with an assumed significance level of 2.5% (one-sided) and a power of 80%, the sample size was calculated to be 69 in each group, with a total of 138 people.

Considering drop-outs, we determined the sample size as 150 subjects in this study.

4.3. Methods

4.3.1. Samples/information used

Of the patients who underwent scheduled surgery under general anesthesia, patients/guardians from whom written consent for participation in this clinical study was obtained and those who were between the ages of 6 months and 65 years on the day consent was obtained were accepted as subjects, with no distinction by sex.

Patients excluded due to safety concerns:

• Patients with myopathy

• Patients with a history of hypersensitivity to rocuronium

• Patients with a history of hypersensitivity to both propofol and thiopental

• Patients with decreased upper limb muscle strength

• Obese patients (BMI ≥30)

4.3.2. Endpoints

Primary endpoint:

The primary endpoint was presence or absence of a withdrawal response after administration of the drugs. Scores from 2 to 4 points on the scale of Ahmad et al., that is, when movement of the arms is seen, were defined as withdrawal response^1^. A score of 1 on Ahmad’s scale, that is, absence of movement, was defined as no withdrawal response.

Secondary endpoints

• Magnitude of withdrawal response assessed using the scale of Ahmad et al.

• Blood pressure before and after drug administration

• Change in heart rate before and after drug administration

4.3.3. Observation and test items

Drug administration method

After entering the operation room, the subjects’ vital signs were monitored, the same as during regular general anesthesia, using an electrocardiograph, pulse oximeter, neuromuscular monitor, and sphygmomanometer.

Anesthesia was induced with intravenous administration of propofol (1-2 mg/kg body weight) or thiopental (3-5 mg/kg body weight). In cases in which standard induction was difficult, anesthesia was induced with inhalation of the anesthetic gas sevoflurane (mixture of 5% concentration in respiratory gas). Loss of consciousness was confirmed by disappearance of the patient’s eyelash reflex, following which the study was commenced. The rocuronium formulation was administered at the dose of 0.9 mg/kg body weight. In the traditional formation group, traditional formula rocuronium was administered, and the novel formula rocuronium was administered in the novel formation group.

Observation of physical changes

The parameters observed were withdrawal response of the arm in which the infusion needle was inserted and changes in heart rate and blood pressure. The arm observations were started just before the administration of rocuronium and made for 3 minutes after its administration. The upper body was imaged with a video camera centered on the arm with the infusion needle, and a research member blinded to the drug content evaluated the withdrawal response as seen in the video camera images and scored it according to its magnitude. The evaluations were done by the same researcher throughout the study to avoid inter-rater bias. Criteria used in previous published reports on vascular pain due to rocuronium injection were employed in the scoring, with a score of 2 or more taken to indicate the presence of a withdrawal response. For the changes in heart rate and blood pressure, the values just before and for 3 minutes after administration were compared with reference to the vital sign monitor records. Observation of the study subjects ended at the point when the 3-minute observations were completed.

Data collection items

The following data items were collected in this study.

• Subject characteristics: Sex, date of birth, height (cm), weight (kg), date consent was obtained, legal representative, primary disease leading to surgery, history of drug hypersensitivity.

• Evaluation at anesthesia: Administered drugs (traditional formation rocuronium, novel formation rocuronium), dosage, administration start date, presence of the arm withdrawal response and magnitude of the response (as evaluated by the blinded research member), heart rate (just before and 3 minutes after administration), systolic and diastolic blood pressure (just before and 3 minutes after administration), TOF ratio according to the muscle relaxation monitor (just before and 3 minutes after administration).

• Adverse events: Type of event, date of occurrence, seriousness, whether there was a causative association, outcome, date outcome was confirmed.

• Drop out information: Day of discontinuation, reason for discontinuation.

4.3.4. Statistical analysis method

The population to be analyzed was taken to be the full analysis set using the assigned treatment group in accordance with the principle of intention-to-treat. Fisher’s exact test was performed for the primary outcome of whether or not there was a withdrawal response. Mantel’s trend test was performed for the secondary outcome of the magnitude of the withdrawal response. Paired t-tests were performed for changes in blood pressure and heart rate before and after drug administration.

For the above analysis method, the details are described in the statistical analysis plan specified at the time of unblinding. However, other statistical method would be used in case that adjusted analysis by using participants’ age or other factors would be preferable.

4.3.5. Drug/medical device overview

Information on the drug used in the trial is summarized below.

Name of drug used: Rocuronium bromide intravenous solution

Study drugs: Traditional formation rocuronium, 50 mg/5.0 mL (MSD K.K.), novel formation rocuronium (Maruishi Pharmaceutical Co., Ltd.)

Approval status in study subjects: Within legal use.

Indications: Muscle relaxation during anesthesia, muscle relaxation during tracheal intubation

Storage: Store at 2–8°C

Clinically significant adverse reactions: Shock, anaphylaxis, prolonged respiratory depression, bronchospasm

Other precautions: There are reports of hearing loss when other nondepolarizing muscle relaxants were administered in the same manner in critically ill neonates or infants.

1. Subject selection

The subjects selected for this study will be patients scheduled for elective surgery under general anesthesia and for whom there is a medical need for the injection of rocuronium. The inclusion and exclusion criteria are mentioned in the previous section (Subjects). A strong withdrawal response after injection of Eslax was clearly demonstrated in minors in previous studies, so the subjects in this study will include juveniles as well as adults.

In this study, legal representatives are defined as follows.

1. A person who has been voluntary appointed as a guardian, a person with parental authority, a guardian, or curator (if applicable).
2. A spouse, adult child, parent, adult sibling, adult grandchild, grandparent, other relative living in the same household, or a person considered to be the equivalent of one of these close relatives.

The following is stipulated for when a legal representative is allowed to give approval in this study.

1. It is objectively judged that the patient is incapable of giving valid informed consent.
2. The patient is a minor. However, the physician in charge will give a full explanation using language that is understandable to the minor patient as well as his or her legal representative, and obtain written consent from both the legal representative and the patient.

Comprehensive assessment of the burden on subjects and the predicted risks and benefits, and measures to minimize the said burden and risk

Comprehensive assessment of the burden and predicted risks and benefits

Burden:

The use of rocuronium formulations is essential when administering general anesthesia, and the disadvantages associated with use of Eslax will not be increased in this clinical trial. In addition, the rocuronium formulation used in this clinical trial is procured using research funds, and will not be an economic burden on the patients themselves.

Risks:

Adverse effects of injection of rocuronium formulation

In a clinical trial in Japan on commercially available Eslax, 32 adverse effects (including abnormal clinical laboratory values) were reported in 18 (3.9%) out of a total of 461 patients. The main adverse effects were fluctuations in clinical laboratory values in 13 cases (2.8%), effects related to heart problems in 3 cases (0.7%), and effects related to vascular problems in 2 cases (0.4%). There is concern that adverse effects will appear at about the same level even with the new drug. However, assuming that a rocuronium formulation will be needed for general anesthesia even without participation in this clinical trial, there would be no increased risk to patients from participation in this trial.

Adverse events associated with observations during the trial

Observation of patients during this clinical trial is limited to the range of observations normally performed during general anesthesia.

Benefit:

Participants in this study will be presented with a gift certificate worth 1,000 yen.

1. Measures to minimize burden and risk

Patients’ conditions will be closely watched during the clinical trial, and utmost care will be taken to minimize the occurrence of adverse events. If an adverse event does occur, it will be dealt with appropriately.

7. Reparation for damage to health, and details of that reparation (in case of study involving invasive procedures)

In case of detrimental health effects in subjects due to adverse effects of the new drug or Eslax injected for this investigation, compensation shall be made using insurance purchased for the study.

8. Handling related to the provision of medical care to study subjects after the study is conducted (in cases when study is accompanied by medical acts that exceed normal treatment)

Patients’ health conditions will be closely observed after completion of the study, and in cases when treatment is necessary it will be provided at our hospital.

9. Evaluation and reporting of adverse events (in cases when study is accompanied by invasive procedures)

Adverse events in this study include all undesirable or unintentional signs, symptoms or diseases that occur during the performance of anesthesia, regardless of whether these events are causally related to the injection of rocuronium formulations.

When an adverse event is judged to have occurred, the name of the event, date of occurrence, severity and outcome will be recorded in the patient’s medical records. When an adverse event occurs, follow-up will be conducted until normalization, recovery, or remission to the level where the condition is no longer considered to be abnormal. Preceding events that continue during performance of anesthesia and events associated with the primary disease will be reported as adverse events only if they are exacerbated during anesthesia.

Serious adverse events in this study are defined as conditions that require hospitalization for treatment, and conditions in which there is impairment or concern of impairment. In cases when a serious adverse event is seen, a judgment will be made as to whether it is a known event described in point 6: “Measures to comprehensively evaluate the burden on subjects, as well as the predicted risks and benefits, and to minimize the said burden and risk,” or whether it is something unknown. When an adverse event occurs, the principal investigator will report it to the university president in accordance with the Kyoto Prefectural University of Medicine Serious Adverse Event Handling Manual.

10. Handling of personal information

The principal investigator (Associate Professor Fumimasa Amaya) will manage subject specimens or other materials by attaching a number (registration number) issued at the time of case registration that is unrelated to the personal information of the subject. When handling specimens or other materials relevant to this study, sufficient care will be taken to protect the confidentiality of the subject (anonymization).

When the correspondence table or other information is managed on a computer, it shall be done with the computer isolated from any network. When the results of the study are made public, they shall not include any information that can be used to identify subjects. Subject specimens and other materials obtained in the study shall not be used for any other purposes.

11. Procedure for receiving informed consent

Obtaining informed consent

A full explanation using the attached explanatory documents will be given to patients during an outpatient visit prior to surgery requiring anesthesia. Written consent is obtained at the discretion of the subject or legal representative. If the patient is 15–19 years old, written consent shall be obtained from both the patient and legal representative. If the patient is under 15 years old and informed consent is obtained from his or her legal representative, the patient’s informed consent will be obtained when it is judged that the subject can express his or her own intention with regard to the study.

If there is any essential change in the objectives or content of the study, the changes will be explained again orally and in writing, and consent will be obtained.

12. Monitoring and auditing system and procedures

Monitoring

In this study, monitoring will be done to ensure that the study is conducted safely and according to protocol, and that data are collected properly. The person in charge of monitoring shall ascertain the state of progress of the trial and confirm compliance with the facility protocol by inspecting the collected patient records. Specific monitoring items and implementation procedures shall be set out in the monitoring procedure manual established before the start of registration.

Auditing

Auditing will not be done in this trial. However, in the event of serious concerns with regard to the study results and ensuring patient safety, the principal investigator shall designate a third party not involved in the trial who can audit the trial.

13. Methods of storing and disposing of specimens and information

Specimens collected and data obtained in this study will be stored appropriately in the Kyoto Prefectural University of Medicine Department of Anesthesiology for 10 years after the results of the study have been published, under the responsibility of Department Professor Teiji Sawa. After that time, they will be disposed of. Anonymized data collected for analysis and for which written consent has been obtained in advance shall be kept after that time for possible use in secondary research (meta-analysis, etc.). In cases when data are used in future research, approval will be obtained again from the Kyoto Prefectural University of Medicine Institutional Review Board.

14. Report content and method of reporting to the head of the research institution

The status of this study will be reported once a year to the university president under the ethics review application system.

If any serious concern arises from the perspectives of respect for the human rights of subjects or others, such as leak of information related to the study, or in terms of implementing the study, a report shall be made immediately to the university president.

15. Conflicts of interest related to studies at the research institution, such as research funding sources, and conflicts of interest related to studies by the researchers and others, such as individual profit

This clinical trial is sponsored by Maruishi Pharmaceutical Co., Ltd., and Fuji Pharma Co., Ltd., who will provide the necessary funding and materials (rocuronium formulations) for the study.

Approval has been obtained from the Kyoto Prefectural University of Medicine Conflict of Interest Committee with regard to the interests of parties in this study. In the event that there is a change in interests, approval shall be obtained after review by the Kyoto Prefectural University of Medicine Conflict of Interest Committee and Kyoto Prefectural University of Medicine Institutional Review Board

16. Method of disclosing information related to this study

This study is registered in the UMIN Clinical Trials Registry, and the content of the study has been disclosed (registration number: UMIN 000022300). An article describing the study results shall be prepared regardless of the nature of the results. The authors shall be selected through discussion between the principal investigator, co-investigators and other related parties, and the first author of the article will be decided. The principal investigator will be the contact person.

1. Shevchenko Y, Jocson JC, McRae VA, Stayer SA, Schwartz RE, Rehman M, et al. The use of lidocaine for preventing the withdrawal associated with the injection of rocuronium in children and adolescents. Anesth Analg. 1999;88(4):746-8.

This protocol was approved by IRB of Kyoto Prefectural University of Medicine (May 19, 2017).
